# Expression and biochemical characterization of a patatin-like medium-chain esterase Rv1063c from *Mycobacterium tuberculosis*

**DOI:** 10.3389/fmicb.2026.1898813

**Published:** 2026-08-19

**Authors:** Yuming Song, Songsong Dong, Rumeng Zhai, Mingli Zhang, Zhengyang Gao, Yufan Jia, Wugeng Han, Hong Lin

**Affiliations:** 1 Weifang Key Laboratory of Respiratory Tract Pathogens and Drug Therapy, School of Life Science and Technology, Shandong Second Medical University, Weifang, China; 2 Clinical Laboratory, Children's Hospital Affiliated to Shandong University, Jinan, China

**Keywords:** *Mycobacterium tuberculosis*, Rv1063c, predicted patatin-like protein, medium-chain esterase, enzymatic characterization

## Abstract

**Introduction:**

*Mycobacterium tuberculosis* is extremely dependent upon lipid-hydrolyzing enzymes to utilize the resources of host lipids and survive within the cell. Rv1063c is a conserved hypothetical protein predicted to share sequence homology with the patatin-like family.

**Methods:**

In the present study, the *rv1063c* gene was cloned and heterologously expressed in Escherichia coli BL21 (DE3). Functional enzyme was obtained by solubilizing inclusion bodies in 8 mol·L^−1^ urea, refolding via gradient dialysis, and purifying through Ni-affinity chromatography.

**Results:**

Bioinformatic homology analysis indicates Rv1063c is homologous to members of the patatin-like family containing a putative Ser52-Asp166 catalytic dyad, though this residue pair has not been experimentally validated in the present study. The recombinant Rv1063c was mainly expressed as inclusion bodies without significant soluble cytoplasmic expression. Biochemical assays demonstrated the recombinant protein exclusively hydrolyzes medium-chain pnitrophenyl esters (C8–12), with maximum hydrolytic activity toward p-NP-C8 (caprylate), followed by C10 and C12, and exhibited the highest activity at 40 °C and pH 7.0. The enzyme exhibited moderate catalytic efficiency but poor thermal stability. All catalytic data in this paper were obtained from artificial p-nitrophenyl ester substrates, which cannot fully reflect natural lipid substrate preference of mycobacteria in vivo.

**Discussion:**

This work biochemically characterizes Rv1063c as a medium-chain-specific esterase encoded by a protein homologous to the patatin superfamily. Its physiological and pathogenic roles remain undetermined, and further multi-level experiments are required to clarify its biological functions.

## 1 Introduction

Tuberculosis (TB) caused by the intracellular pathogen, *Mycobacterium tuberculosis* (Mtb), remains one of the leading causes of human death worldwide due to the effects of a single infectious agent (World Health Organization, 2025). Although there has been a continuous development in antibiotic treatment and vaccine programs, the development of multidrug resistance, latent disease, and HIV co-infection remain as barriers to the global eradication of TB (Corbett et al., 2003). One of the characteristic aspects of Mtb pathogenesis is its extraordinary ability to hijack and metabolize the lipids of the host, alter cellular membrane components and establish long-term persistence within host macrophages (Russell, 2001; Almeida et al., 2023). These results have led to the widespread recognition of lipid metabolism and lipid-hydrolyzing enzymes as master regulators of mycobacterial physiology, cell wall integrity, stress adaptation and virulence (Lin et al., 2024).

Lipolytic enzymes—comprising lipases, esterases, phospholipases, and lysophospholipases—participate in a broad spectrum of physiological processes, including lipid catabolism, signal transduction, membrane dynamics, and nutrient acquisition (Gupta et al., 2004). In mycobacteria, these enzymes are instrumental in degrading host-derived triacylglycerols, phospholipids, and fatty acid esters, a capacity that underpins intracellular persistence and immune evasion (Menon et al., 2025). Patatin-like proteins have attracted considerable attention among lipolytic enzyme families due to their conserved catalytic motifs and broad substrate promiscuity across diverse organisms (Wilson and Knoll, 2018).

Patatin-like family hydrolases contain the conserved GXSXG lipase motif and a Ser-Asp catalytic dyad (Rydel et al., 2003). Members of this superfamily have diverse catalytic capacities reported in different species, including esterase, lipase and phospholipase activities (Kienesberger et al., 2009). Various patatin-like lipases in Mtb have been associated with the halt of phagosome maturation, intracellular survival and host cell apoptosis regulation (Parker et al., 2009; Cui et al., 2020). However, many hypothetical patatin-like proteins remain functionally uncharacterized, representing a critical knowledge gap in mycobacterial lipid metabolism (Ragavendran et al., 2022).

The *rv1063c* gene is annotated in the *M. tuberculosis* H37Rv genome as encoding a conserved hypothetical protein. Although bioinformatic analysis assigns it to the patatin-like family and predicts a lipolytic function, its catalytic activity, substrate profile, optimal reaction conditions, and mechanistic basis have thus far eluded experimental scrutiny. Characterization of this enzyme thus contributes to the fundamental understanding of mycobacterial lipid metabolism. A well-recognized challenge in the heterologous production of mycobacterial proteins is their propensity to aggregate as inclusion bodies in *Escherichia coli*, with soluble expression representing the exception rather than the norm (Mitraki et al., 1991; Carrió and Villaverde, 2000). In accordance with this trend, Rv1063c was undetectable in the soluble cytoplasmic fraction, nevertheless, active enzyme was successfully recovered through solubilisation, purification, and refolding of the inclusion-body material. Following cloning and expression, the recombinant protein was subjected to comprehensive biochemical characterization, encompassing substrate specificity, reaction optima, stability, and kinetic behavior. Our results establish that recombinant Rv1063c functions as a medium-chain-specific esterase, a finding consistent with its predicted patatin-like structural features.

## 2 Materials and methods

### 2.1 Bioinformatic and structural analysis

The full-length nucleotide sequence and amino acid sequence of Rv1063c was retrieved from the Mycobrowser database (https://mycobrowser.epfl.ch/genes) (Camus et al., 2002). Sequence similarity analysis was performed using the NCBI BLAST tool (Altschul et al., 1990). Multiple sequence alignment was carried out with BioEdit v7.0.1 (Hall, 1999). A phylogenetic tree was constructed via the neighbor-joining algorithm in MEG4 (Kumar et al., 2004).

For structural modeling, a homology model of Rv1063c was generated using SWISS-MODEL, with the crystal structure of the patatin-like protein PLPD from *Pseudomonas aeruginosa* (PDB: 5fya.1) serving as the template (Waterhouse et al., 2018). Visualization and structural analysis were performed in YASARA (Krieger et al., 2002). Conserved motifs and putative active-site residues were identified through sequence alignment and structural comparison (Rydel et al., 2003). The sequence identity between Rv1063c and the template was 33.59%, SWISS-MODEL quality metrics (GMQE = 0.56, QMEAN = 0.61) indicated a reliable model.

### 2.2 Bacterial strains, plasmids, and reagents

The heterologous protein expression host strain was *Escherichia coli* BL21(DE3). The pET28a vector carrying an N-terminal His-tag was used for gene cloning and protein expression (Hochuli et al., 1988). *M. tuberculosis* H37Ra genomic DNA was used as PCR template (Cole et al., 1998). The rv1063c coding sequence of *M. tuberculosis* H37Ra shares 100% nucleotide identity with the H37Rv reference genome deposited in Mycobrowser. DNA sequencing of our cloned PCR fragment further verified full sequence consistency with H37Rv rv1063c without any mutation.

Kanamycin (50 μg mL^−1^ final concentration) was supplemented in LB medium for transformant screening. PrimeSTAR Max DNA Polymerase, *Eco*R I, *Hin*d III, T4 DNA ligase, DNA and protein markers were purchased from Takara (Dalian, China). Oligonucleotide synthesis and Sanger sequencing were completed by Tsingke Biotechnology (Qingdao, China). A series of p-nitrophenyl fatty acid esters were obtained from Sigma-Aldrich (St. Louis, MO, USA). All other chemical reagents were of analytical grade and commercially available.

### 2.3 Cloning of the *rv1063c* gene

The full-length *rv1063* coding sequence was amplified by PCR using *M. tuberculosis* H37Ra genomic DNA as template (Cole et al., 1998). Forward primer: 5′-CGGAATTCGATGCCCGCACCAGCTG- 3′ (*Eco*R I site), Reverse primer: 5′-CCCAAGCTTGCCCTCGATGGTCGCAGC- 3′ (*Hin*d III site) (Hochuli et al., 1988).

The 50 μL PCR system contained 25 μL 2 × PrimeSTAR Max Premix, 1 μL forward primer, 1 μL reverse primer, 0.2 μL template DNA and 22.8 μL sterile ddH_2_O. Thermal cycling parameters: initial denaturation at 98 °C for 5 min; 25–30 cycles of 98 °C 10 s, 60 °C 5 s, 72 °C 1 min; final extension at 72 °C for 7 min. PCR products were separated on 1.0% agarose gel and purified using a DNA gel extraction kit.

### 2.4 Construction of recombinant expression plasmid

Purified *rv1063c* PCR fragment and empty pET28a vector were double-digested with *Eco*R I and *Hin*d III (Hochuli et al., 1988). Each 50 μL digestion system contained 1 μL each restriction enzyme, 5 μL 10 × Buffer K, corresponding DNA template and sterile water, incubated at 37 °C for complete digestion. Digested vector and insert were ligated in a 20 μL system (1 μL T4 ligase, 2 μL 10 × ligase buffer, 5 μL linearized vector, 12 μL insert DNA) at 16 °C overnight. Ligation product was transformed into competent cells, spread on kanamycin-containing LB agar plates. Positive colonies were validated by colony PCR and DNA sequencing (Sanger et al., 1977).

### 2.5 Expression of recombinant Rv1063c protein

*E. coli* BL21(DE3) colony carrying pET28a-*rv1063c* was cultured overnight at 37 °C, 220 rpm in 5 mL kanamycin-supplemented LB medium. Overnight culture was diluted 1:100 into fresh LB medium and cultured to OD_600_ = 0.6–0.9. Protein expression was induced with 1 mmol·L^−1^ IPTG at 25–30 °C for 16 h with shaking to reduce inclusion body formation (Hochuli et al., 1988). Cells were harvested by centrifugation at 8,000 × g, 4 °C and stored at −80 °C.

### 2.6 Purification of recombinant Rv1063c

Cell pellets were resuspended in lysis buffer (20 mmol·L^−1^ Na_2_HPO_4_-NaH_2_PO_4_, pH 7.0, 20 mmol·L^−1^ imidazole, 500 mmol·L-1 NaCl). After sonication and centrifugation, all target protein was found in the insoluble fraction, with no detectable soluble supernatant. Inclusion bodies were solubilised in 8 mol L^−1^ urea (Mitraki et al., 1991), and the solubilised supernatant was loaded onto a pre-equilibrated Ni-NTA HisTrap HP column (1 mL, GE Healthcare, Little Chalfont, Buckinghamshire, UK) (Bornhorst and Falke, 2000). Non-specific bound proteins were washed off until the *A*_280_ baseline stabilized, and the target protein was eluted with a linear 20–500 mmol L^−1^ imidazole gradient (Porath, 1992). Eluted fractions were analyzed by SDS–PAGE. Refolding was accomplished by gradient dialysis against buffers containing successively decreasing urea concentrations (4, 2, and 0 mol·L^−1^), each supplemented with 20 mmol·L^−1^ phosphate (pH 7.0), 0.1 mmol L^−1^ EDTA, 10% (*v/v*) glycerol, and 0.01% Triton X-100. Each dialysis step lasted 12 h at 4 °C, with buffer replacement every 6 h. Four negative controls were included to ascertain the specificity of enzymatic activity: (i) blank refolding buffer without recombinant protein, (ii) purified protein from cells harboring empty pET28a vector, (iii) heat-inactivated rRv1063c (95 °C, 10 min), and (iv) unrefolded protein in 8 mol·L^−1^ urea. The concentration of refolded protein was determined by the Bradford (1976) assay, using BSA as the standard.

### 2.7 SDS–PAGE and western blot analysis

12% separating gel and 5% stacking gel were used for SDS–PAGE (Laemmli, 1970). Protein samples were mixed with 5 × loading buffer and heated before electrophoresis. Gels were stained with Coomassie Brilliant Blue R-250 (Laemmli, 1970). Separated proteins were transferred to PVDF membranes (Towbin et al., 1979). Membranes were blocked with 5% skim milk, incubated with anti-His primary antibody and HRP-conjugated secondary antibody, then visualized by ECL chemiluminescence (Burnette, 1981).

### 2.8 Circular dichroism spectroscopy for conformational verification

Proper folding of the refolded rRv1063c was verified by far-UV CD spectroscopy using the 8 mol·L^−^^1^ urea-denatured protein as a negative control. Samples were buffer-exchanged into urea-free 20 mmol·L^−^^1^ phosphate (pH7.0) and diluted to 0.15 g·L^−^^1^ (Bradford assay), then spectra were recorded over 190–260 nm on a Jasco J-815 spectropolarimeter (0.1 cm cuvette, 25 °C) with a scan speed of 50 nm·min^−^^1^, bandwidth of 1 nm, and response time of 1 s under N_2_ purge. After subtracting the buffer blank and averaging three replicate scans, raw ellipticity (mdeg) was converted to mean residue ellipticity (MRE, × 10^−^^3^ deg·cm^2^·dmol^−^^1^) using [*θ*] = (*θ*_*obs*_ × 100)/(*C* × *l* × *N*), where *θ*_*obs*_ is the measured ellipticity, *C* is the protein concentration (g·L^−^^1^), *l* is the path length (cm), and *N* is the number of amino acid residues (360).

### 2.9 Enzymatic activity assay

Lipolytic activity was determined spectrophotometrically using p-nitrophenyl caprylate (p-NP-C8) as reference substrate (Kordel et al., 1991). All reactions were performed in a total volume of 200 μL. The reaction mixture contained 20 mmol·L-1 phosphate buffer (pH7.0), 0.5 mmol L^−1^ p-NP-C8 and appropriate enzyme solution, The mixture was incubated at 40 °C for 5 min, and absorbance at 410 nm (*A*_410_) was subsequently measured. p-Nitrophenyl substrates were dissolved in acetonitrile, with final acetonitrile ≤ 5% *v/v* in reaction system. Exact volume of purified refolded enzyme was fixed in all reaction systems. Independent p-nitrophenol standard curves were generated under each test pH to eliminate pH-dependent absorbance bias. One unit (U) of activity was defined as the enzyme amount releasing 1 μmol p-nitrophenol per minute under assay conditions. All activity measurements were performed in three parallel experimental replicates using the same batch of purified refolded protein. Mean ± standard deviation (SD) was calculated and displayed as error bars in all activity figures.

### 2.10 Biochemical characterization of Rv1063c

Substrate specificity was assessed with a series of p-nitrophenyl fatty acid esters varying in acyl chain length: C4 (butyrate), C8 (caprylate), C10 (caprate), C12 (laurate), C14 (myristate), and C16 (palmitate). Each reaction mixture (200 μL total volume) contained 20 mmol·L^−1^ phosphate buffer (pH 7.0), 0.5 mmol·L^−1^ of the respective substrate, and 50 μL purified, refolded rRv1063 protein. Specific activities (U·g^−1^ total protein) toward each substrate were calculated independently. All activity measurements were carried out with three parallel experimental replicates prepared from a single batch of purified refolded protein.

Optimum temperature was measured at 25–55 °C (Lin et al., 2019). Thermal stability was determined by measuring residual activity after incubation at 40 °C at different time points, *ln*(% residual activity) vs. time was fitted to first-order decay to calculate kd and *t*_1/2_ = *ln*2/*k*_d_ (Shu et al., 2016). Optimum pH was tested in 20 mmol·L^−1^ buffers: NaAc-HAc (pH 4.0–5.0), phosphate (pH 6.0–8.0), Gly-NaOH (pH 9.0). pH stability was determined by 12 h incubation at 4 °C across pH gradients (Shu et al., 2016). Independent p-nitrophenol standard curves were generated individually at each tested pH (4.0–9.0). Briefly, 10 mmol^−1^ p-nitrophenol acetonitrile stock was serially diluted with matching pH buffers to obtain concentration gradients from 0 to 1.0 mmol^−1^. Each standard mixture was adjusted to a total volume of 200 μL with acetonitrile proportion kept under 5%, identical to the enzyme reaction system. All standard samples were incubated at 40 °C for 5 min following the same incubation protocol used in activity tests. *A*_410_ absorbance of each gradient was recorded in triplicate, with buffer blanks as baseline for each pH. Linear standard equations were fitted for every pH condition separately. The separated calibration curves corrected pH-dependent shifts in p-nitrophenol absorbance and avoided quantitative errors in activity calculation.

Kinetic constants *K*_m_ and *V*_max_ were determined with p-NP-C8 concentrations of 0.05–0.95 mmol·L^−1^, fitted to Michaelis–Menten model via non-linear regression in GraphPad Prism 8 (GraphPad Software, San Diego, California, USA). *k*_*cat*_ = *V*_max_/[*E*], where [*E*] is molar enzyme concentration (Zhao and Arnold, 1999). The molar concentration [*E*] was converted from Bradford-determined total protein mass concentration via the formula: [*E*] = *C*/*M*, where *C* represents protein mass concentration (g·L^−^^1^) measured by Bradford assay, and *M* denotes the theoretical molecular mass of His-tagged rRv1063c (43,000 g·mol^−1^, 43 kDa). Control experiments confirmed >98% activity originates from target protein, negligible non-enzymatic background. All fitted kinetic parameters were reported with standard error (SE).

All assays were conducted with three independent biological replicates, and three technical replicates were included per biological replicate. One-way ANOVA followed by Tukey's *post-hoc* test was applied to assess significant differences in activity across temperatures, pH and substrates.

## 3 Results

### 3.1 Sequence and structural analysis of Rv1063c

**Figure 1:**
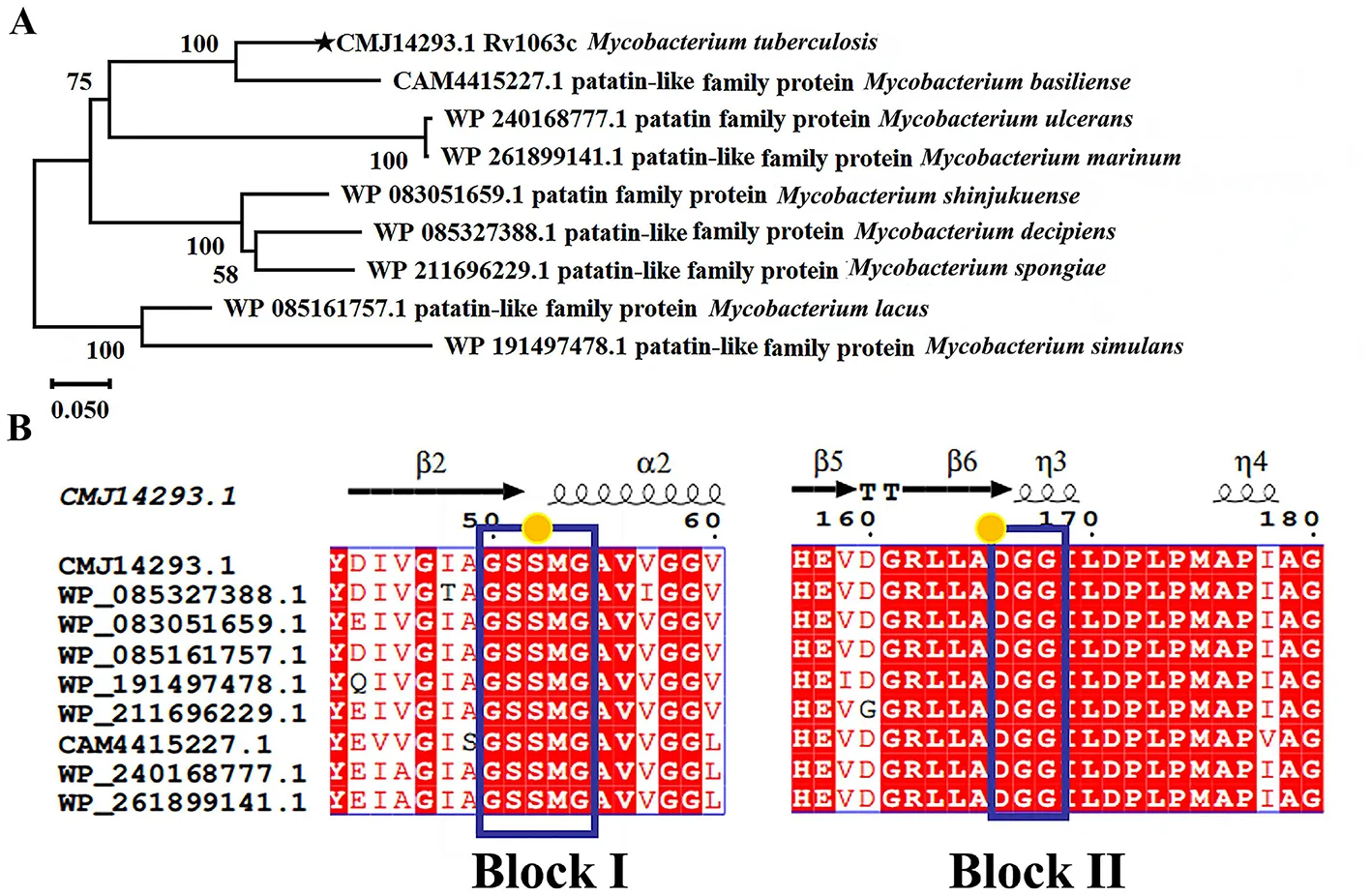
Bioinformatic analysis of Rv1063c. **(A)** Phylogenetic tree of Rv1063c and homologous predicted patatin-like family proteins of several mycobacterial strains. Neighbor-joining method with 1000 bootstrap replicates. **(B)** Multiple sequence alignment. Conserved Block I (Gly50-X-Ser52-X-Gly54) and Block II (Asp166-Gly168) are boxed in blue, putative catalytic Ser52 and Asp166 are labeled. All figures contain error bars representing mean ± SD of three biological replicates.

The *rv1063c* gene from *M. tuberculosis* H37Ra encodes a 360-amino-acid polypeptide with a theoretical molecular mass of 37.5 kDa (Cole et al., 1998; Camus et al., 2002). Homology analysis supports its assignment to the patatin-like protein family (Rydel et al., 2003; Kienesberger et al., 2009) (Figure 1A). Multiple sequence alignment revealed two conserved motifs—Gly50-X-Ser52-X-Gly54 (Block I) and Asp166-Gly168 (Block II)—which are signature features of this superfamily (Figure 1B). Phylogenetic analysis showed that Rv1063c clusters closely with mycobacterial patatin homologs, with the highest homology to patatin-like family protein in *Mycobacterium basiliense*, indicating strong evolutionary conservation.

**Figure 2:**
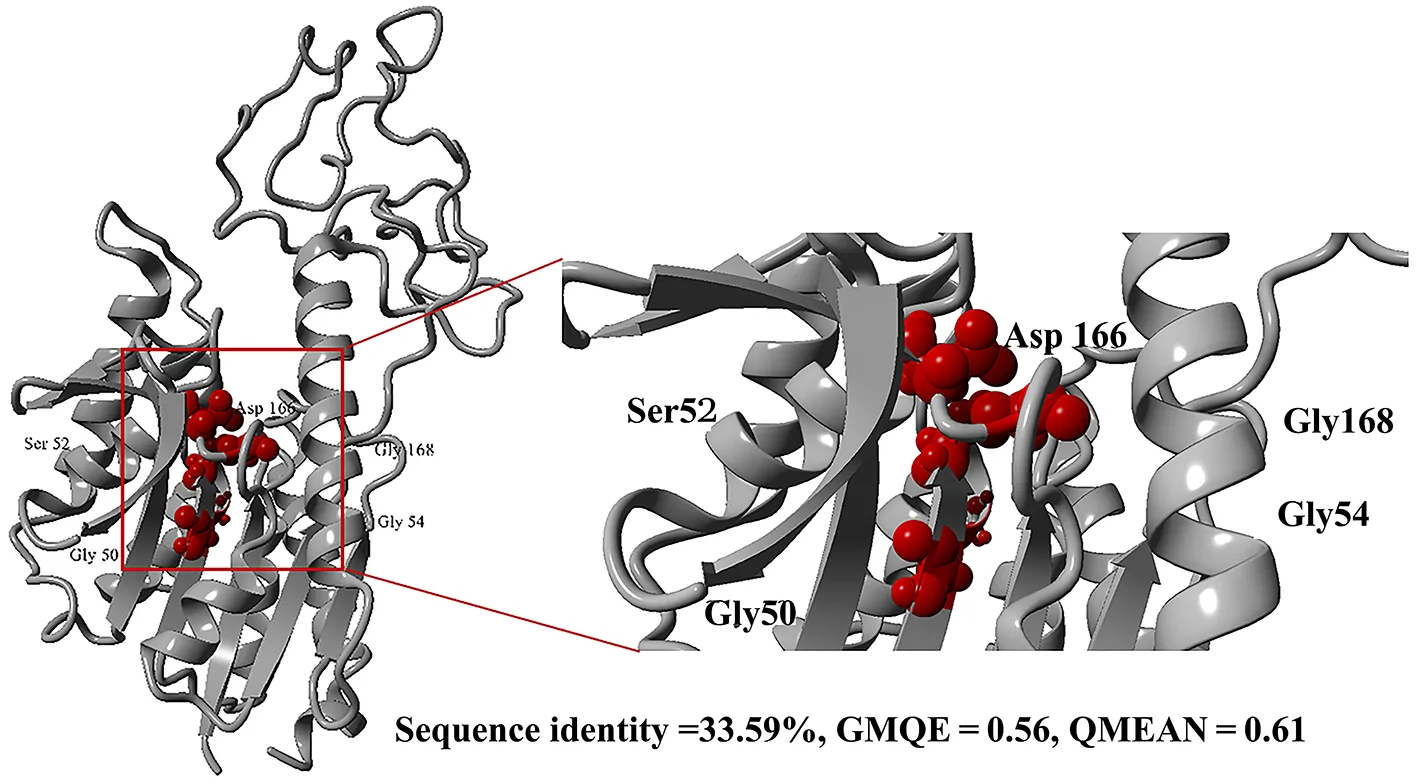
Homology model of Rv1063c built on PDB 5fya.1 template. Arrows: β-strands; spirals: α-helices. Conserved Gly50, Ser52, Gly54, Asp166, Gly168 shown as red spheres. Ser52 and Asp166 are proposed to form a putative catalytic dyad based on homology modeling, without experimental verification via site-directed mutagenesis.

Homology modeling was built using *P. aeruginosa* PLPD (PDB: 5fya.1) as template, presenting a canonical α/β hydrolase fold containing 11 α-helices, 8 β-strands and five 3_10_ helices (Figure 2). Homology structural modeling further supports the hypothesis that Ser52 and Asp166 constitute a putative catalytic dyad potentially mediating hydrolytic activity, however, mutagenesis experiments are required to validate their catalytic function.

### 3.2 Cloning, expression, and purification of recombinant Rv1063c

**Figure 3:**
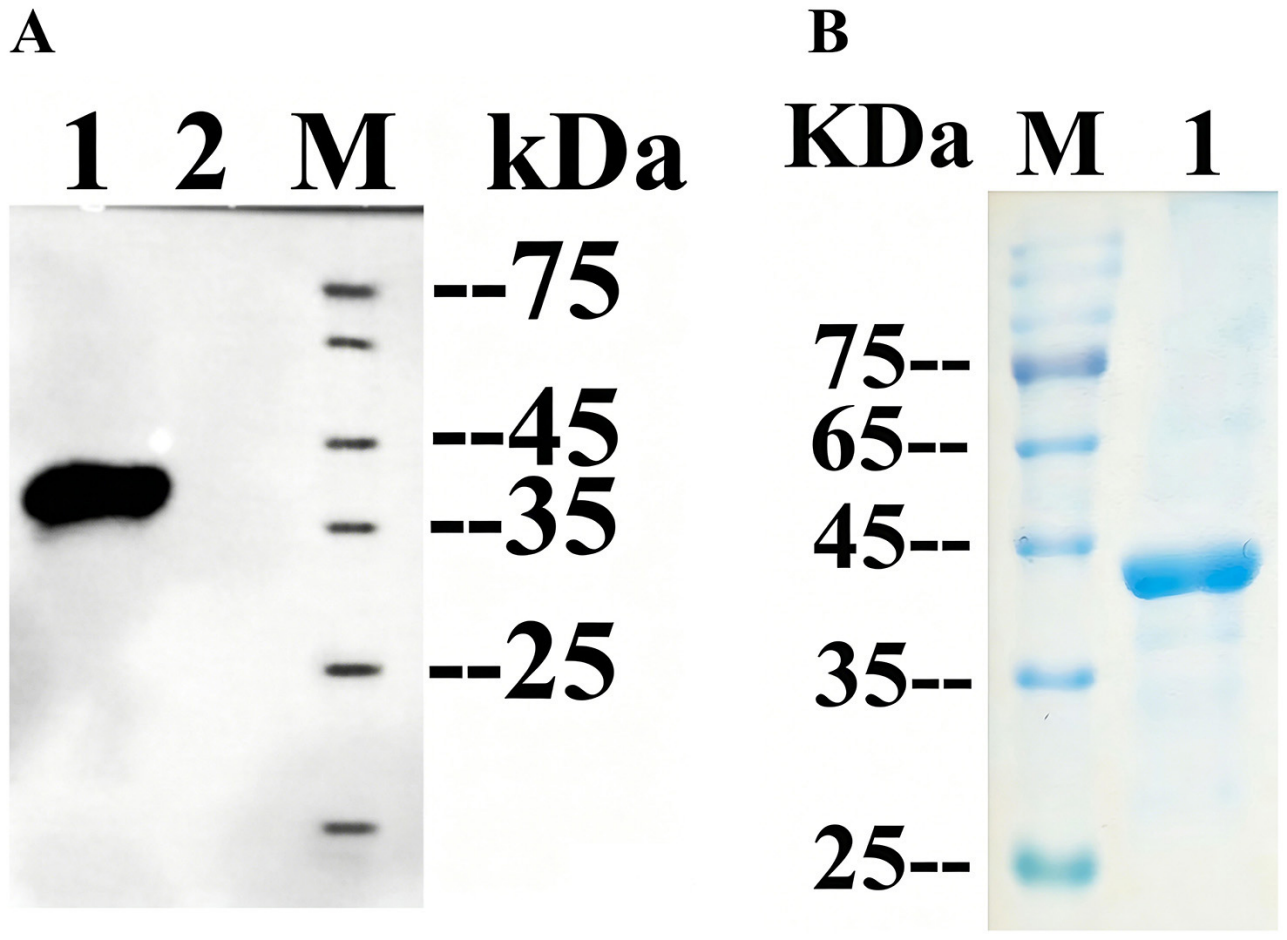
Expression and purification analysis of rRv1063c. **(A)** Western blot identification. M, protein molecular weight marker; lane 1, inclusion body fraction; lane 2, soluble supernatant fraction. Immunoblot result confirmed that recombinant rRv1063c was predominantly expressed as inclusion bodies. **(B)** SDS–PAGE analysis of purified refolded rRv1063c. M, protein molecular weight marker; lane 1, purified target protein at approximately 43 kDa. A faint low-molecular-weight minor band was detected; this minor fragment accounted for only 3.8% of total protein and barely interfered with activity quantification.

The full-length rv1063c open reading frame was amplified by PCR, double-digested, and inserted into pET28a to yield pET28a-rv1063c, the correctness of which was verified by Sanger sequencing. Induction with 1 mmol·L^−1^ IPTG at 25–30 °C for 16 h resulted in abundant production of rRv1063c, which accumulated exclusively as inclusion bodies. No soluble protein was detected in the supernatant after sonication (Figure 3A). Multiple soluble expression attempts were tested, including induction at 16 °C for 24 h, 0.1 mmol·L^−1^ low-concentration IPTG. However, no detectable soluble recombinant Rv1063c(rRv1063c) was obtained under all tested conditions. The inclusion bodies were dissolved in 8 mol·L^−1^ urea, purified by Ni-affinity chromatography, and refolded by gradient dialysis to recover enzymatic activity. The final concentration of refolded protein was 0.77 g·L^−^^1^, and the overall recovery of active protein was 12.43 ± 1.12% of the total inclusion-body protein. Enzymatic activity assays demonstrated that the dialyzed refolded rRv1063c possessed catalytic function, preliminarily indicating successful protein refolding. Four negative controls, including refolding buffer without recombinant protein, empty pET28a vector-derived purified protein, heat-inactivated rRv1063c (95 °C, 10 min), and unrefolded protein dissolved in 8 mol·L^−1^ urea, yielded no significant hydrolytic signals, verifying the specificity of its enzymatic activity.

SDS–PAGE showed a single dominant band at approximately 43 kDa, consistent with the theoretical mass of the His-tagged fusion protein (Figure 3B). Densitometry analysis of SDS–PAGE gel showed the target His-tagged rRv1063c band accounted for 96.2% of total protein. A faint low-molecular-weight minor band was observed below the target band. The proportion of this fragment was extremely low, so it exerted negligible interference on all enzymatic activity measurements.

**Figure 4:**
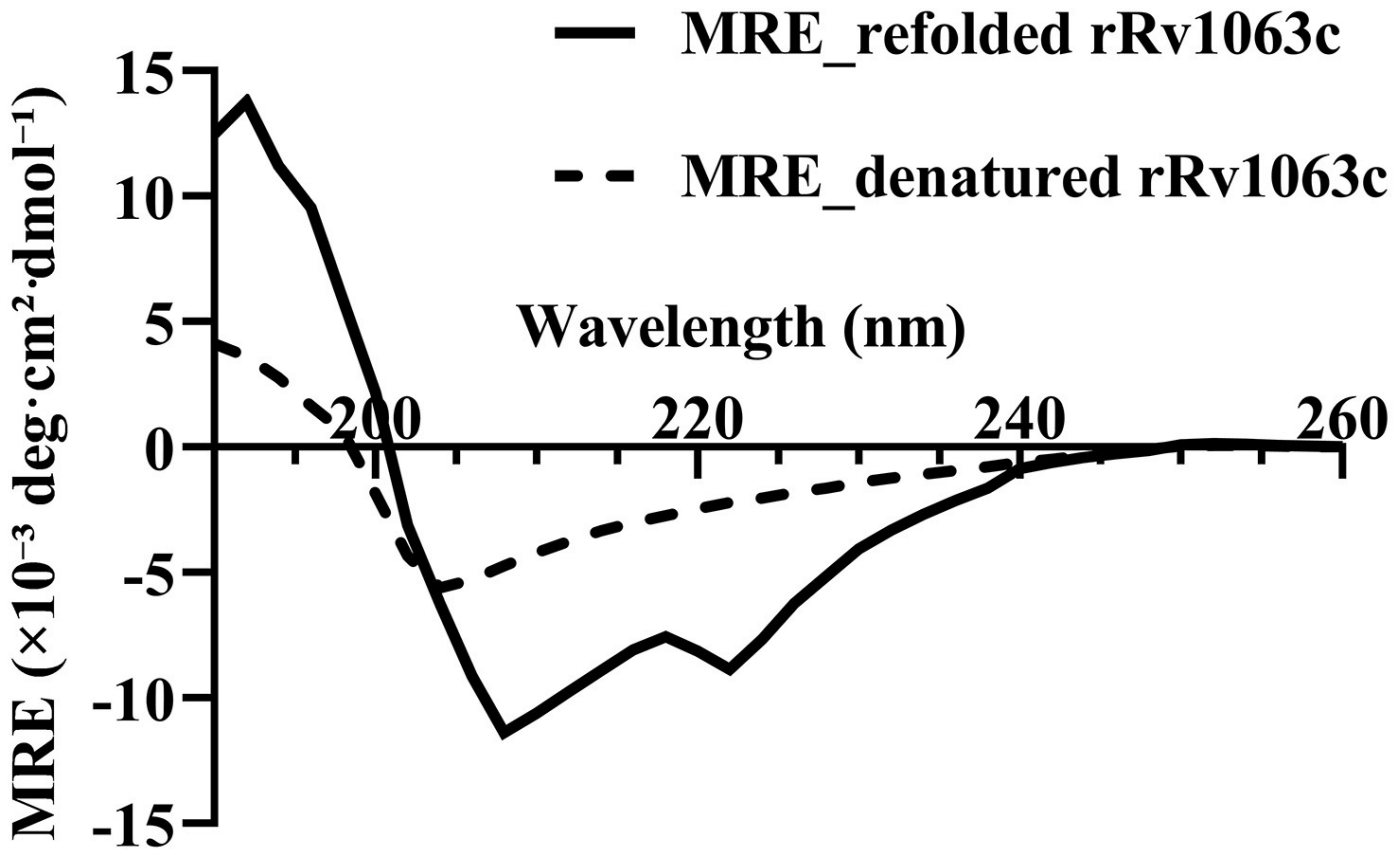
Far-UV CD spectra of refolded rRv1063c (solid black line) and 8 mol·L^−1^ urea-denatured rRv1063c (dashed gray line). *Y*-axis: mean residue ellipticity scaled by 10^−^^3^ deg·cm^2^·dmol^−^^1^.

Circular dichroism (CD) spectroscopy was performed to directly characterize the secondary structure of refolded rRv1063c. The CD spectrum of refolded protein showed canonical α/β hydrolase characteristics: a strong positive peak at 192 nm, two distinct negative troughs at 208 nm and 222 nm, and a weak negative shoulder at ~217 nm corresponding to β-sheet components. In contrast, the 8 mol·L^−^^1^ urea denatured control only displayed a broad negative peak near 200 nm without helical signals. The spectral difference between folded and denatured protein confirmed that gradient dialysis restored ordered globular conformation without massive protein aggregation (Figure 4).

### 3.3 Substrate specificity of recombinant Rv1063c

**Figure 5:**
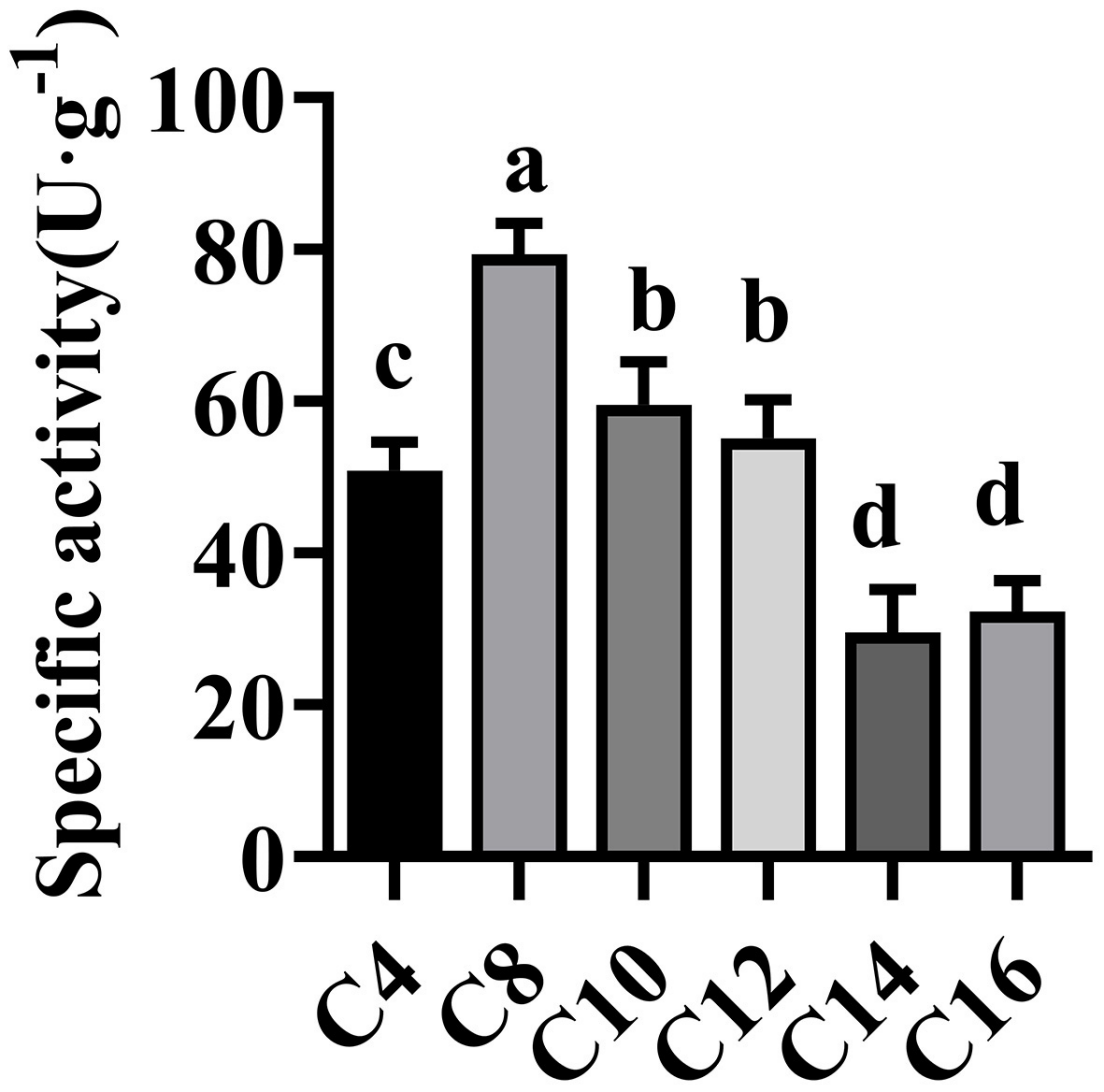
The substrate specificity of recombinant rRv1063c. Absolute specific activity of refolded rRv1063c toward p-nitrophenyl esters with acyl chains ranging from C4 to C16 (C4: pNP-butyrate, C8: pNP-caprylate, C10: pNP-capricate, C12: pNP-laurate, C14: pNP-myristate, C16: pNP-palmitate). Values are mean ± SD of three biological replicates. Different lowercase letters denote significant differences (one-way ANOVA, Tukey's test, *p* < 0.05).

The substrate-specificity data demonstrated that rRv1063c preferentially hydrolyses medium-chain p-nitrophenyl esters. The highest activity was recorded against p-NP-C8 (caprylate), with a specific activity of 79.36 ± 4.08 U·g^−1^, followed by C10 (caprate) and then C12 (laurate). Weak activity was detected against the long-chain C14 and C16 substrates (Figure 5). This pattern defines rRv1063c as a medium-chain-specific esterase on synthetic substrates. It should be emphasized that p-nitrophenyl fatty acid esters are only chromogenic artificial substrates for rapid enzyme screening, and their binding pocket environment differs greatly from endogenous mycobacterial lipids. The medium-chain preference observed here cannot be extrapolated to its actual *in vivo* substrate spectrum. The natural substrate spectrum of Rv1063c requires further exploration.

### 3.4 Optimal temperature and thermal stability of recombinant Rv1063c

**Figure 6:**
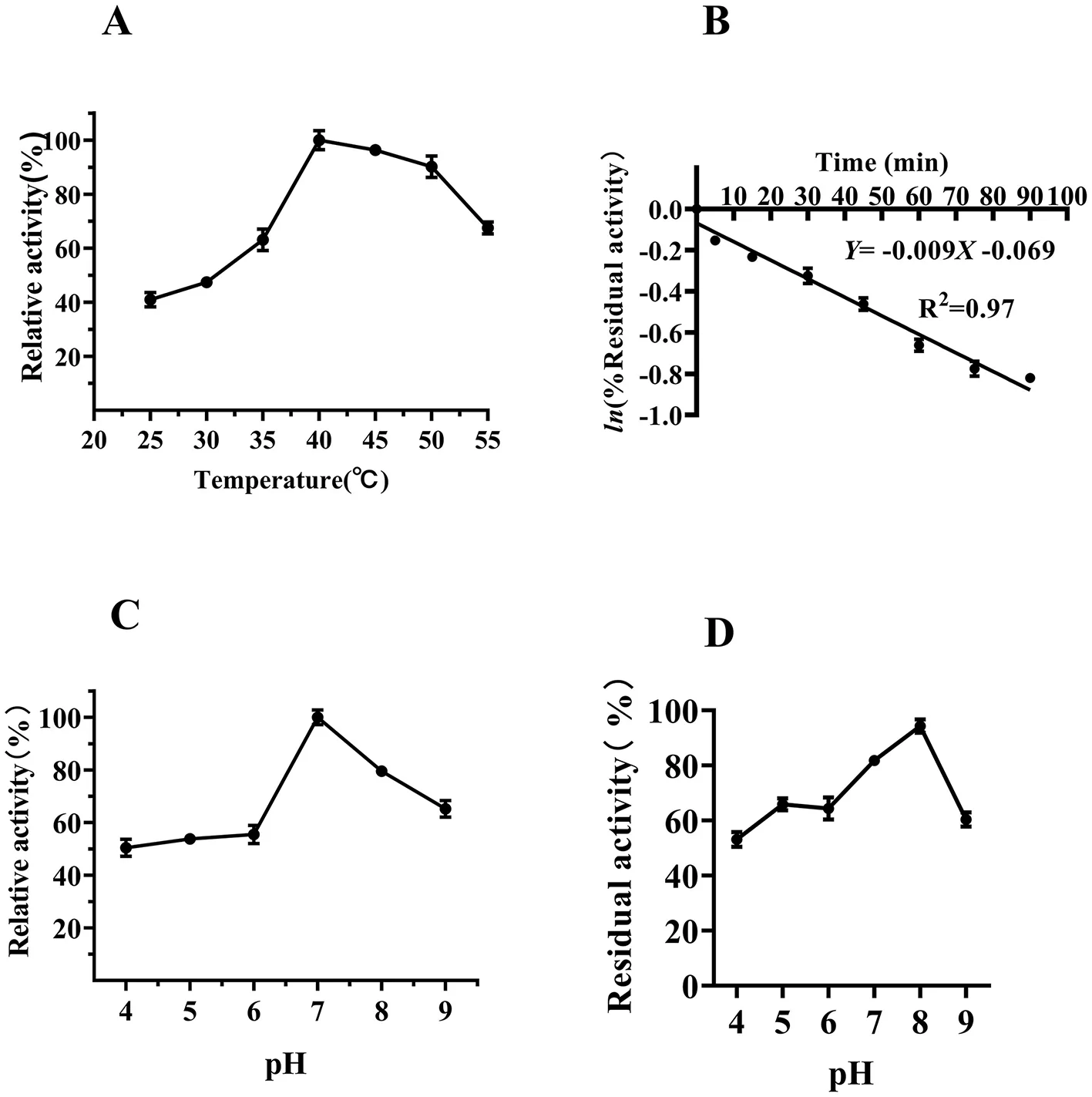
Biochemical characterization of refolded rRv1063c under different temperature and pH conditions. **(A)** Optimal temperature. The highest catalytic activity was observed at 40 °C. **(B)** Thermal stability. The enzyme maintained stable activity below 40 °C and was thermally inactivated at higher temperatures. **(C)** Optimal pH. The enzyme exhibited maximum activity at pH 7.0 and decreased sharply under acidic and alkaline conditions. **(D)** pH stability. rRv1063c remained stable at neutral pH but lost activity under extreme pH conditions. Values are mean ± SD of three biological replicates.

The recombinant enzyme exhibited maximal activity at 40 °C (defined as 100% relative activity), with a gradual decline above this temperature and a marked loss above 45 °C (Figure 6A). Upon incubation at 40 °C, residual activity decreased steadily over time, yielding a thermal inactivation half-life (*t*_1/2_) of 62.75 min, indicative of poor thermostability (Figure 6B). This value is considerably shorter than that reported for the characterized mycobacterial patatin homolog Rv3802c (*t*_1/2_ > 120 min at 37 °C) (Parker et al., 2009), further reinforcing the conclusion that rRv1063c is relatively thermolabile compared to related mycobacterial lipolytic enzymes.

### 3.5 Optimal pH and pH stability of recombinant Rv1063c

The enzyme displayed maximal catalytic activity at pH7.0, with a steep decline under acidic conditions ( ≤ pH 6.0) and negligible activity between pH 4.0 and 5.0 (Figure 6C). When incubated for 12 h at 4 °C across a range of pH values, the enzyme retained approximately 95 % of its activity at pH8.0 and about 80 % at pH 7.0, whereas activity dropped sharply at pH ≤ 6.0 and was moderately reduced at pH 9.0 (Figure 6D). Collectively, these results indicate that rRv1063c exhibits greater stability under near-neutral to weakly alkaline conditions (pH 7.0–8.0) than in strongly acidic environments.

### 3.6 Kinetic parameters of recombinant Rv1063c

**Figure 7:**
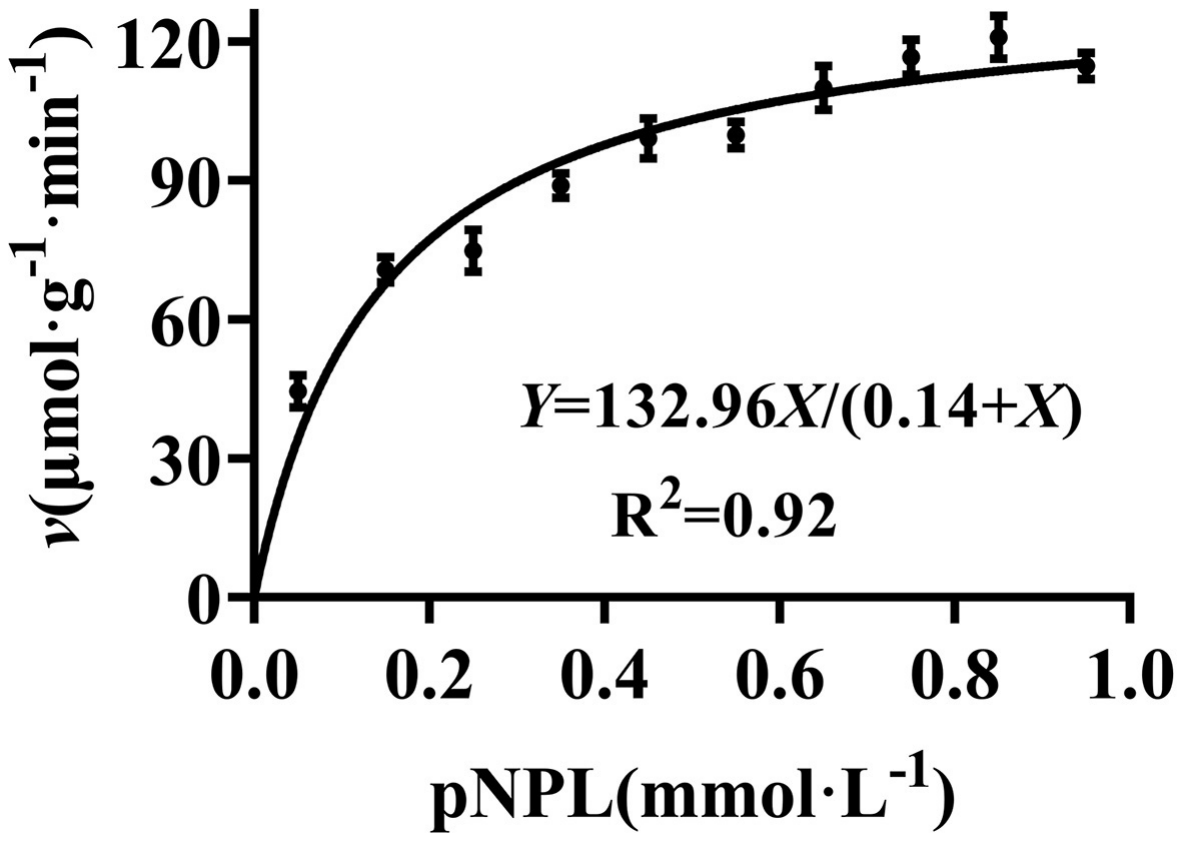
Michaelis–Menten kinetic curve of refolded rRv1063c against p-NP-C8 substrate. The hyperbolic curve was fitted by non-linear regression following the equation *Y* = 132.96*X*/(0.14 + *X*) with *R*^2^ = 0.92, yielding kinetic constants *K*_m_ and *V*_max_ together with their fitting standard errors (SE). The observed trend conforms to typical substrate-saturable Michaelis–Menten enzymatic kinetics. Data are presented as mean ± SD from three biological replicates.

Kinetic parameters were determined using p-NP-C8 at concentrations spanning 0.05–0.95 mmol·L^−^^1^ (Figure 7). Non-linear regression of the data to the Michaelis–Menten equation yielded the following values (with standard errors): *K*_*m*_ = 0.14 ± 0.02 mmol·L^−^^1^, *V*_*max*_ = 132.96 ± 3.95 μmol·g^−^^1^·min^−^^1^, *k*_*cat*_ = 95.29 ± 2.83 s^−^^1^, and *k*_*cat*_/*K*_*m*_ = (6.81 ± 0.99) × 10^5^ L·mol^−^^1^·s^−^^1^. These kinetic constants suggest that rRv1063c possesses moderate substrate affinity and catalytic efficiency toward its preferred medium-chain ester substrate.

## 4 Discussion

Tuberculosis remains a global public health crisis, and mycobacterial lipid metabolism is essential for intracellular survival and virulence (Lin et al., 2024; World Health Organization, 2025). Among lipolytic enzymes, patatin-like proteins attract broad attention due to unique catalytic architecture and diverse biological functions (Wilson and Knoll, 2018; Cui et al., 2020). This study reports the first systematic cloning, expression, purification and biochemical characterization of unannotated hypothetical protein Rv1063c. We experimentally verified that Rv1063c functions as an active medium-chain-specific esterase, sequence and structural homology analyses place it within the patatin-like family protein.

Bioinformatic analysis identified the conserved motifs Gly50-X-Ser52-X-Gly54 and Asp166-Gly168, which are canonical signatures of the patatin-like family protein (Rydel et al., 2003; Kienesberger et al., 2009). Multiple alignment and phylogenetic clustering with other mycobacterial patatin homologs suggest evolutionary conservation and potentially analogous biological roles (Krieger et al., 2002). Homology modeling, based on the *P. aeruginosa* PLPD structure (PDB: 5fya.1), supports a putative catalytic dyad composed of Ser52 and Asp166. The overall fold conforms to the canonical α/β hydrolase architecture, consistent with its patatin-like classification. Nevertheless, the actual catalytic contribution of these residues remains unconfirmed, and the proposed mechanism is speculative in the absence of site-directed mutagenesis data.

Heterologous expression in *E. coli* BL21(DE3) gave rise almost exclusively to inclusion bodies—a commonly encountered difficulty for mycobacterial proteins, often attributable to their high hydrophobicity and the lack of appropriate mycobacterial chaperones in the *E. coli* host (Altschul et al., 1990; Mitraki et al., 1991). Despite this aggregation, we successfully obtained functional rRv1063c by urea solubilisation, Ni-affinity purification, and gradient-dialysis refolding. SDS–PAGE and western blotting confirmed a purified product of ~43 kDa, matching the theoretical mass of the His-tagged fusion. To eliminate the doubt that enzyme activity may originate from aggregated misfolded protein, we supplemented far-UV CD characterization according to standard protocols for patatin-family hydrolases. The spectral features of refolded rRv1063c were consistent with the typical α/β fold of PlpD (Hanson et al., 2024), whose crystal structure was used as our homology modeling template. The obvious difference between folded and denatured spectra, together with quantitative secondary structure fitting, provides direct spectral evidence that our dialysis refolding strategy successfully generated properly folded recombinant protein, so all subsequent enzymatic data are reliable.

The substrate screening revealed that rRv1063c selectively degrades medium-chain p-NP esters (C8–C12), with the highest activity on C8 and much lower activity on long-chain (C14/C16) substrates. This distinct medium-chain preference sets rRv1063c apart from classical lipases (which favor long chains) and short-chain esterases. Given that this preference was only determined using artificial chromogenic substrates, we cannot draw any definitive conclusions regarding the natural lipid substrates utilized by Rv1063c inside mycobacterial cells.

The optimal temperature of 40 °C is close to human febrile temperature. Further experiments are needed to clarify the biological significance of this thermal property. However, the enzyme displayed poor thermostability, with a half-life of only 62.75 min at 40 °C, implying that *in vivo* chaperones or membrane associations may be required for stabilization, transiently controlled lipid metabolism may be a crucial feature, consistent with other thermolabile microbial lipases (Shu et al., 2016; Lin et al., 2019).

Activity was maximal at pH 7.0, whereas storage stability peaked at pH 8.0, and residual activity dropped sharply below pH 6.0. The neutral pH optimum matches the cytoplasmic and early phagosomal pH of mycobacteria, suggesting that rRv1063c primarily functions in near-neutral intracellular compartments and may not be active in the acidic environment of mature phagolysosomes, thus distinguishing its functional niche from that of acid-tolerant mycobacterial lipases. The kinetic parameters obtained with p-NP-C8 indicate moderate catalytic efficiency, implying that Rv1063c is a specialized medium-chain esterase rather than a broad-spectrum hydrolase, and that it likely participates in selective lipid catabolism rather than general nutrient acquisition.

This study functionally annotates Rv1063c as a patatin-homologous medium-chain esterase with neutral pH preference and poor thermostability. At present, we only obtained enzymatic data from artificial substrates, there is no experimental evidence to support its physiological or pathogenic functions inside mycobacteria or host cells.

Several lines of investigation remain to be pursued to clarify the functional relevance of Rv1063c. Site-directed mutagenesis of the predicted catalytic residues Ser52 and Asp166, combined with quantitative activity assays, is needed to confirm their roles in catalysis. In parallel, mycobacterial gene knockout and complementation assays would provide insight into its physiological contributions to growth and stress adaptation. The identification of endogenous lipid substrates is also critical for understanding its native function, while the establishment of more efficient soluble expression systems would support future structural studies and the discovery of interacting partners.

## 5 Conclusions

Taken together, this work delivers the first comprehensive biochemical and structural characterization of *M. tuberculosis* Rv1063c. Sequence homology and structural modeling only show Rv1063 shares conserved motifs with the patatin-like family protein. Although a Ser-Asp dyad is predicted, we have no experimental proof of catalytic function, and only medium-chain esterase activity was verified with synthetic substrates. Following heterologous expression in *E. coli* (with the protein accumulating predominantly in inclusion bodies), active recombinant protein was recovered by denaturation, Ni-affinity purification, and dialysis-based refolding. Substrate hydrolysis assays verified that rRv1063c acts as a medium-chain-specific esterase, with a clear preference for C8–C12 p-nitrophenyl esters. All activity data in this paper rely on artificial chromogenic ester substrates. The enzyme displays maximal activity at 40 °C and pH 7.0, with moderate catalytic efficiency but weak thermal stability. These biochemical features only reflect the catalytic property of Rv1063c toward synthetic ester substrates. Overall, this study completes preliminary biochemical detection of recombinant Rv1063c and expands basic knowledge of mycobacterial patatin-homologous proteins. All physiological functions of Rv1063 remain to be verified via mycobacterial genetic and cellular experiments.

## Data Availability

The original contributions presented in the study are included in the article/supplementary material, further inquiries can be directed to the corresponding author.
